# Claude Bernard and life in the laboratory

**DOI:** 10.1007/s40656-023-00570-x

**Published:** 2023-03-21

**Authors:** Hans-Joerg Rheinberger

**Affiliations:** Max Planck Institute for the History of Science, Berlin, Germany

**Keywords:** Claude Bernard, Physiological laboratory, Experimental practice, In vivo Experimentation, Science and Art

## Abstract

Much has been written on Claude Bernard as a relentless promoter of the experimental method in physiology. Although the paper will touch Bernard’s experimental intuitions and his experimental practice as well, its focus is slightly different. It will address the laboratory, that is, the *space* in which experimentation in the life sciences takes place, and it will analyze the scattered remarks that Bernard made on the topic both in his books and in his posthumously published writings. The paper is divided into four parts. The introduction briefly sketches the coming into being of the physiological laboratory in the first half of the nineteenth century. The second section will give an overview of Claude Bernard’s own itinerary in physiology and his personal laboratory experience. The third part of the paper will have a look at the image of the laboratory that Claude depicted in his *Introduction to Experimental Medicine*. In the subsequent section and by contrast, the image of the laboratory will come into focus as it can be reconstructed from Bernard’s notebook that he kept between 1850 and 1860, the *Cahier rouge*. Finally, the fifth part of the paper will spotlight Claude Bernard’s comparison of the sciences and the arts and their respective practices. A brief concluding statement tries to summarize Bernard’s epistemological position toward experimentally practiced science.

## Introduction: the coming into being of the physiological laboratory

Physiological laboratories properly speaking are an invention of the second half of the nineteenth century. They became only gradually established along with the process of the institutional separation of physiology from anatomy that European universities underwent in the second third of that century (Schiller, 1968). In his book *Wissenschaft in der Maschinenstadt. Emil Du Bois-Remyond und seine Laboratorien in Berlin*, Sven Dierig (2006) has described the coming into being of a physiological laboratory in detail and situated it in the context of nineteenth century industrialization and urbanization. In the first decades of the nineteenth century, medical students specializing in physiological questions usually still had to conduct their experiments – if they experimented at all — in a corner of their private homes and chambers. Dierig has aptly called this phase “chamber physiology” – “*Stubenphysiologie*” (Dierig, 2000). In the second third of the century, anatomists pursuing physiological questions, such as, e.g., Johannes Müller in Berlin, who had followed anatomist Karl Asmund Rudolphi on his chair at Berlin’s University in 1833, and who was also in charge – as it then used to be — of the anatomical collection, had to find a space in the collection rooms, if he aimed at doing experiments besides his teaching duties (Lohff, 1993; Otis, 2007). It is only in the last third of the century that physiologists started to be endowed with institutes suited for their special purposes, of which the “Physiologische Anstalt” of Carl Ludwig in Leipzig was one of the first and most richly equipped (Lenoir, 1997). It opened its doors in 1869 and became a paradigm of a physiological working space for Europe as well as for overseas, especially the United States.^1^ In his later addresses, Claude Bernard did not miss to point to these developments in neighboring Germany, claiming that his own country would be the legitimate place to house such institutions, being the country in which modern physiology had originated. In his course on general physiology that he pronounced from 1869 to 1876 at the Muséum d’Histoire Naturelle, where he had succeeded Marie-Jean-Pierre Flourens after the latter’s death in 1868, and that later appeared as the famous *Leçons sur les phénomènes de la vie communs aux animaux et aux végétaux*, he gave the following brief description of Ludwig’s lab: “I put before your eyes the plan of one of these laboratories, that of Leipzig directed by Ludwig,^2^ which is sketched here in the beautiful report of M. Wurtz:^3^ I wished that on this example you can see the richness of these scientific installations, of which we do not even have an idea in France. In the basement there are the cellars, the rooms kept at constant temperature, the distillation apparatuses, a steam engine that sustains movement everywhere, the atelier of a mechanic attached to the laboratory, a magazine for the chemical products, a hospital for the dogs. – In the first floor there are the laboratories of vivisection, those of physics and biological chemistry, the chambers for handling mercury, the rooms for the microscopes, for the histological studies, for the spectroscope, and so on. – The library, the rooms for the courses, the lodging of the professor, are part of the same building; add a horse stable, an aviary, numerous aquaria, and we have enumerated the essential parts of this magnificent establishment erected for science” (Bernard, 1966, pp. 15–16). And in the report on general physiology that he wrote on the instigation, in 1867, of Victor Duruy, then Minister of Public Instruction, he stated: “One can see that, for instance, if Germany has the biggest share in contemporary publications in the science of physiology, this is a result of the fact that the cultural means of experimental physiology are considerable and well instituted there.” And he did not forget to add: “French physiology reclaims nothing else than what is only fair to give it; it never lacked the physiological genius” (Bernard 1965a, pp. 210–211). However, Paris had to wait until 1880 for its first full-fledged state-supported physiological laboratory to be built in town for Etienne-Jules Marey who had taken over the chair of Pierre Flourens at the Collège de France in 1869.

## Claude Bernard’s physiological itinerary and his personal laboratory experience

Claude Bernard’s first laboratory experience dates back to 1840, when he entered the Collège de France as an assistant and subsequently became “préparateur” to François Magendie in 1841. Magendie had been appointed professor for physiology and general pathology at the Collège de France in 1836. He had managed to install a modest laboratory at the Collège, the first and only laboratory space of physiology in France at the time. Paul Bert, a student of Bernard and his successor on the chair for general physiology at the Sorbonne, on the occasion of Bernard’s death in 1878, once succinctly voiced this turning point in Bernard’s life as follows: “As soon as he had put his foot in the laboratory at the Collège de France, his path was traced. The famous physiologist’s [Magendie’s] intrepid, albeit a bit disordered, experimentation, his implacable critique, his skepticism that included his own discoveries, made a deep, creative impression on the spirit of the young Claude Bernard” (Bert, 1911, p. 17).

It is here that over the course of the following decade Bernard developed his own style as a physiological experimenter. In his magisterial work on *Claude Bernard and Animal Chemistry*, Frederic Holmes (1974) has meticulously followed, on the basis of his early laboratory notebooks, the “investigative pathway” of Bernard that led him, among other things, to the discovery of the glycogenic function of the animal liver. Bernard did not have a circumscribed research question to begin with. Rather, his early steps in the laboratory appear as “a bit disordered” like those of his teacher, to use the wording of Paul Bert. Among the questions he approached were animal combustion, a theme virulent in physiology since the heroic days of Antoine Lavoisier. With respect to the latter, in his late *Leçons* still, Bernard confessed: “The discovery of respiratory combustion by Lavoisier has been, one can say, more fruitful than the majority of anatomical discoveries” (Bernard, 1966, p. 7). Furthermore, efforts to determine the nutritive value of gelatin were imposed on Magendie’s laboratory by a commission of the government that sought to determine cheap nutrients that were hoped to be able to replace the expensive meat nutrition in Parisian hospitals and asylums. He worked on dynamic aspects of blood circulation as well as on the uptake and the course of drugs through the body. And not least, his interest was attracted by the decomposition of nutrients, such as sugars, in the animal’s organs (Holmes, 1974; Rheinberger, 2013, Ch. 6).

It was an amalgam of questions on which Bernard tried his hands in these early years, without conspicuous results to begin with. The ensemble of trials was, however, held together by the conviction that if one wanted to know what was specifically physiological, or biological, about these processes, one had to experiment on and with the living animal body. This conviction was to be condensed, in the course of the 1850s, into his notion of “milieu intérieur” (d’Hombres, 2015/2016). This went against the grain of physiological chemistry of the time, as particularly represented by Jean-Baptiste Dumas in France and Justus von Liebig in Germany that was essentially based on the chemical analysis of metabolic products, a comparison of inputs and outputs of sorts. Bernard was not at all opposed to chemical analysis; on the contrary, he frequently sought the help of chemists for secondary analysis of materials he had recovered from his experimental animals. However, he held on to the maxim that it was foremost necessary to identify the intermediate products of metabolism that were specifically formed in the living body’s internal environment. Accordingly, he sought to develop an arsenal of procedures that would allow him to introduce substances into the body in a localized manner and to retrieve substances such as gastric juice in a similarly localized way and according to a particular time regime. He thus established what became known as in vivo experimental physiology. This first decade of his experimental activities led him to the unsettlement of a deep-seated dogma of contemporary physiology: the conviction that the synthesis of organic materials was restricted to plants, whereas their decomposition was the privilege of animals. He had started out, among other strands of work he pursued, to analyze the decomposition of sugar in the animal body; what he ended up with was an investigation of the opposite: the synthetic glycogenic function of the liver.

I think that we can draw two lessons from Claude Bernard’s laboratory regime as it developed at the Collège de France in the course of the 1840s, and that would also guide Bernard’s experimental efforts in later years (Grmek, 1973, 1991). The first is that Bernard developed a style of experimentation that we can address as specifically exploratory.^4^ As the laboratory notebooks of the time between 1840 and 1850 document (Holmes, 1974, 2003), to stay with this example, Bernard conducted several lines of experimentation in parallel. He developed what Gerald Geison, in a review of Holmes’ book on *Claude Bernard and Animal Chemistry*, called “Bernard’s real methods”, contrasting them with the “idealized prescriptions for scientific research” laid down in his *Introduction to the Study of Experimental Medicine* (1865) (Geison, 1975, p. 639). He thus could explore the range of action of the experimental gadgets and devices that he had developed, and he could adapt and modify them accordingly. He could also use the findings of one series of experiments and implement them, if possible and feasible, in another one. In this way, he was able to develop an experimental network that again and again led him to surprising revelations. He was also ready, if with the actual state of knowledge and equipment he ended up in an impasse, to temporarily abandon a particular stream of experimentation and switch to another one, until a new finding or a novel experimental device allowed him to switch over again to the one he had put aside for the time being. It is an experimental strategy that works, so it appears, particularly well if a new field of research is in the process of being opened and delineated. The history of the sciences knows of a number of variants of that strategy (Rheinberger, 2000; Holmes, 2004). Bernard himself, at the end of his career, summarized it in the following words: “In order to tackle experimental criticism and to get to know all the conditions of a physiological phenomenon, one must have groped for a long time, one must have been deceived a thousand times, one must have, in a word, grown old in experimental practice” (Bernard, 1966, p. 19).

The second lesson is that Bernard came to be convinced that minutiae matter and that it is decisive to develop a sensorium for them. In the introduction to his classic published in 1865, the *Introduction*, he put it as follows: “In scientific investigation, the most minute procedures are of the highest importance. The lucky choice of an animal, an instrument construed in a specific manner, the use of one reagent instead of another, will often suffice to resolve general questions of a highest order. […] In one word, the greatest scientific truths have their roots in the details of experimental investigation that constitute in some sort the soil in which these truths develop.” And he concluded: “One must have been brought up and have lived in laboratories in order to grasp the full importance of all these details of procedure in investigation that are often ignored and despised by those false scholars who call themselves generalizers” (Bernard, 1984, p. 44).

This feeling for the details, the attention to the contingencies going along with the intricacies of experimentation was a lesson that Bernard learned early on and that he held in highest esteem throughout his later career. In his Preface to the *Introduction*, François Dagognet has summarized Bernard’s attitude toward experimentation and his receptivity with regard to the empirical detail as follows: “Theory, in turn, may play the role of a springboard as well as that of an obstacle. One discovers less with ideas than against them, because the learned scholar must become a “doubter” who tries to understand the language of nature beyond the interpretation that, aiming to reveal it, also disguises and confuses it. Yes, one must question life, but one has to be attentive above all to the answers it gives in the margins or even outside the expected discourse” (Dagognet, 1984, p. 18). Bernard walked these margins, and they revealed to him their riches over and over again.

Throughout his life, Bernard had to work under precarious laboratory conditions. Since the beginning of the 1840s, he had François Magendie’s modestly equipped laboratory at the Collège de France at his disposal. Before that time, Magendie himself had to give “private courses in experimental physiology,” as Bernard euphemistically called these sessions. And he recollects: “It is only after 1830 that, having become professor of medicine at the Collège de France, he was able to establish the largely insufficient laboratory that there still exists […]” (Bernard, 1966, p. 10). The list of people with whom Bernard cooperated throughout the 1840s, among them Jean Poiseuille, Jean-Baptiste-Rozier Coze, and Charles-Louis Barreswil, amply demonstrates that he depended on the equipment of others, above all chemists, in particular for the secondary analysis of the bodily products that he retrieved from his in vivo experiments (Holmes, 1974). The situation did not much improve when he became, in 1855, the successor of Magendie. “After having become successor of Magendie at the Collège de France, I have fought like him against the lack of resources. […] Back then, the laboratory at the Collège de France was the only one that existed” (Bernard, 1966, p. 11). No laboratory facilities were attached to the chair of general physiology at the Sorbonne either, to which Bernard had been appointed in 1854. Consequently, he later moved to the chair left vacated by the death of Pierre Flourens at the Muséum d’histoire naturelle in 1868, where he could finally install another laboratory of physiology in Paris. There, he proudly introduced his opening lecture in the summer semester of 1870 with the following words: “The introduction of general physiology in the renowned establishment that houses the natural sciences, and the creation of a laboratory annexed to the chair mark a notable progress in teaching experimental physiology.” And he looked back, not without reproach to the responsible authorities: “This completely modern science that originated in France under the fruitful impulse of Lavoisier, Bichat, Magendie, etc., was until now, it must be said, left practically without encouragement, whereas in the neighboring countries, in contrast, it received considerable sustainment” (Bernard, 1966, p. 1). And then, he undergirded the importance of laboratory work, not only for teaching, but in particular for research: “Finally, the majority of scientific questions are resolved by the invention of an adequate tool: he who discovers a new procedure, a new instrument, often achieves more for experimental physiology than the deepest-minded philosopher or the most powerful generalizer” (Bernard, 1966, p. 12).

## The laboratory in the “Introduction to experimental medicine”

With a few interspersed exceptions, Bernard’s personal laboratory experience remains in the background of the *Introduction to Experimental Medicine*. Overall and in general, the *Introduction* depicts a rationalized image of the laboratory process at large and of experimentation in particular, an image that is in accordance with contemporary philosophy of science. The papers that Bernard left document that he studied, excerpted, and commented upon, among others, the philosophy of Auguste Comte, one of the leading positivist philosophers of the time (Bernard, 1954). Bernard operates with the traditional categories of induction and deduction, proof and counter-proof, of truth and error, hypothesis and fact, determinism and indeterminism. I will not add here to the abundant literature about the *Introduction*. It is one of the rare cases where historians, philosophers and scientists crossed paths and shared their reflections on the modern sciences again and again.^5^ Instead, I will restrict myself to Bernard’s remarks on the specificities of a physiological laboratory.

The second part of the *Introduction* contains a long chapter devoted to experimental considerations particularly concerning the manipulation of living animals. As already said, Bernard founded his way of practicing physiology on experimentation in vivo. It was clear for him that physiology had to use the means and procedures of physics and of chemistry, if only “with a great number of inherent difficulties” (Bernard, 1984, p. 145) for he assumed that the physico-chemical processes, i.e. their “determinism,” were the same in the non-living and in the living world. On the other hand, there was something irreducibly special about living beings: “In one word, biology has its own problem and its specific point of view; it only makes use of the help and borrows the methods from the other sciences, but not their theories” (Bernard, 1984, p. 144). Bernard stayed with this non-reducible difference and its epistemological consequences, which he came to attribute to the organism’s peculiar “internal organic environment,” to which life was inextricably linked (Bernard, 1984, Part II, Chap. 1, § 3). His position gave rise to an endless quarrel about whether and if, to what extent, he can be regarded as a “vitalist” (Virtanen, 1960; Canguilhem, 1967, 1968a, b; Bange, 2019).

I do not pursue this debate here; rather, what appear to me to be important are two things that connect to his vivisectionist approach. The first point is that Bernard was convinced that this non-reducible difference, that is, the specific way organisms made use of the physico-chemical determinism, as he called it, could only be grasped in the living body itself. He had a hunch that the specificity of the living body’s internal environment hung together with its use of ferments, of which, however, knowledge was still scarce in the 1860 and 1870s. “We have thus to consider, besides the physico-chemical conditions indispensable for the manifestation of life, the special evolutionary physiological conditions which are the *quid proprium* of the biological science” (Bernard, 1984, p. 149).^6^ Experimentation in vivo appeared thus as a logical consequence of this conviction. The second point, neither to be neglected, is that Bernard was working in the tradition of medical physiology, that is, with the human body in mind. Experimentation on humans, and on the living human body in particular, was, however, utterly restricted. Experimentation on higher animals, as practiced by Bernard, thus appeared as a viable option to get as near as possible to the human body in terms of experimental analysis.

Keeping this reasoning in mind, it is not by chance if Bernard claimed that an adequate physiological laboratory had to be of a complex nature. “The laboratory of the medical physiologist must be the most complicated of all laboratories, since he has to experiment on the phenomena of life, which are the most complex of all natural phenomena” (Bernard, 1984, p. 199). In his later *Lectures on the Phenomena of Life Common to Animals and Plants*, and being now offered the opportunity to establish a laboratory of his own, he differentiated this claim further and specified it at the same time: “The laboratory of the physiologist is necessarily complex in view of the complexity of the phenomena that are studied here. It has naturally to be arranged for three different orders of work: 1. the work of *vivisection*; 2. the *physico-chemical* works; 3. the *anatomical-histological* works” (Bernard, 1966, p. 16).

The internal structure of the physiological laboratory was not the only point of concern for Bernard, however. Another point of attention and reflection was the relation between the laboratory of the experimental physiologist and the clinic. He took care not to confound the two: “The hospital, or better, the ward, is not the laboratory of the physician, as is often believed; the latter is, as we have said already, only his field of observation […]” (Bernard, 1984, p. 205). And he explains this relation further: “The physician who is eager to merit this designation in a scientific sense must, having left the hospital, go into his laboratory, and it is here that he must try, by way of experiments on animals, to account for what he has observed on his patients, be it with respect to the mechanism of the illness, be it with regard to the action of drugs, or be it concerning the origin of the morbid lesions of the organs or tissues” (Bernard, 1984, p. 206).

And finally, Bernard does not forget to count the library among the knowledge spaces between which the practitioner of scientific medicine has to move. “The libraries could also be considered to form part of the laboratory of the scientist and the medical experimenter.” But he immediately adds his *caveat*: “This is, however, only the case if he reads in order to know and control by nature the experiences or theories of his predecessors, and not in order to find in the books the ready-made opinions that would dispense him from working […]” (Bernard, 1984, p. 199). The working environment of the experimental physiologist is thus tripartite in a double sense. Internally, there is a partition and circulation of knowledge between vivisection, physico-chemistry, and anatomy/histology. And the experimental laboratory itself is part of another triple: the laboratory, the clinic, and the library.

## The laboratory in Bernard’s *Cahier rouge*

If we understand, with Mirko Grmek (1965), Bernard’s laboratory notes between 1850 and 1860,^7^ also called the *Cahier rouge*, as a part of his intellectual preparation for the composition of his later book on *Experimental Medicine*, we can nevertheless observe an opposite tendency. Whereas in the *Introduction*, Bernard positions himself as a theoretician, or philosopher, or epistemologist of the experiment; in the *Cahier rouge*, he writes as a practitioner immersed in his daily work. Whereas in the *Introduction*, he generally follows what could be called an ‘idea first’ protocol, in the *Notebook*, an ‘idea follows observation’ protocol prevails. It can of course be argued, and rightly so, that all the elements of Bernard’s discourse on experimentation to be identified in the notebook can be found in the introduction as well. It all depends, however, on the relative weight attributed to them in the unpublished notes and in the published *chef d’oeuvre*. As a rule, in the *Introduction*, Bernard avoids, with a few exceptions, to point to what he calls “groping experiments” (*expériences de tâtonnement*) or “experiments for the sake of seeing if” (*expériences pour voir*) and qualifies them as being inferior and belonging to “a science in its infancy” (Bernard, 1984, p. 50). The general tenor of the book, however, is characteristically captured by the following succinct and unequivocal, reassuring statement: “It is the idea that constitutes, as we shall see, the starting point or the *primum movens* of all scientific reasoning, and it is the idea that is likewise the goal in the aspiration of the scientific spirit toward the unknown” (Bernard, 1984, p. 56).

In the *Cahier rouge*, whose character conveys something like the intimacy of a laboratory confession not destined for a public readership, the tendency is the other way around. Here, the poetological aspect of the laboratory prevails (Rheinberger, 1999; Sattar, 2013). What is presented in a tamed form and between the lines in the *Introduction*, here it finds its spontaneous expression. But this also implies that from the perspective of the *Notebook*, we can read the *Introduction* with new eyes.

In his introduction to the *Cahier rouge*, Mirko Grmek has rightly stressed the double face of these notes. On the one hand, they contain reflections that Bernard himself subsumed under the heading “scientific philosophy.” On the other hand, the majority of entries consist of spontaneous considerations concerning experiments to be made. Mostly, the two kinds of entries do not appear to be directly related to each other. They are likely to be the result of what Bernard himself described with the following words in the *Cahier*: “The ideas develop themselves spontaneously in the mind, and if one lets oneself go with them, one is like a man at the window who regards people passing […]. It does not require any effort; it is even charming” (Bernard 1965b, p. 89). Grmek has concluded that “we are confronted with a thought that is in the process of being formed, and not a finished thought” (Grmek, 1965, p. 13). And he summarized: “The experimental protocols let us see how the laboratory of Claude Bernard functioned; the book bound in a red envelope, on its part, allows us to enter into the interior laboratory of his thoughts” (Grmek, 1965, p. 17).

Let us first give a brief impression of Bernard’s remarks on what he calls “scientific philosophy.” Under the heading “Ideas to develop,” we can read: “[…] State that in fact one never makes a discovery by looking for it directly […] Science proceeds by way of revolutions, not by way of pure and simple additions […]” (Bernard 1965b, p. 149). Accordingly, he describes his own scientific itinerary as follows: “I came to the field of science on a devious route, and I have rid myself of rules by launching myself across fields, something that others would probably not have dared to do. But I think that in physiology this has not been bad, because it has led me to new insights” (Bernard 1965b, pp. 128–129).

Consequently, in these notes, Bernard sings the praises of ignorance. The following statement sounds like a confession: “I am not a materialist. – I am not a vitalist either. – The vitalists claim; the materialists claim the opposite. – I say: I claim nothing, I know nothing; it’s the truth, and it is this state of ignorance in which I am that allows me to make hypotheses, to poetize, to indulge in my feelings and to follow my nature” (Bernard 1965b, p. 118). Later on in the *Notebook*, he stresses once again: “There is a certain pleasure in ignoring, because then the imagination can work” (Bernard 1965b, p. 157). One is reminded here of Bernard’s later notebook *Philosophie*, which ends on the following note: “If [man] needs to know, he no less needs to ignore in order to aim at knowing. If man knows everything, he will be annihilated. As Pascal says, man is made for searching after the truth and not for its possession” (Bernard, 1954, p. 43). The first task of the experimenter appears thus to be keeping oneself open for the unexpected.

After glossing over some of these more general reflections, insofar as they concern life in the laboratory, let us now have a brief look into the parts of the *Notebook* that concern themselves with Bernard’s sketches of experiments to be pursued or carried out in the future. Most conspicuously, they reveal to us an aspect of Bernard’s laboratory regime, or style of experimentation that he obviously practiced over and over again. We could, for want of a better expression, call it experimenting as difference making, differential experimentation, or searching for robust differences.^8^ We could also call it comparative experimentation, as Bernard himself does later in the *Introduction* under the title “Comparative Experimentation” (Bernard, 1984, pp. 181–185). A quick overall count reveals at least a dozen places in the *Cahier rouge*, where Bernard conceived of and vividly described experiments directed at exposing differences that he hoped would serve as the starting points for further experimentation. Let us have a brief look at one of them, which Bernard put under the heading of “comparative digestion.” Here we read: “In order to know the role of the intestinal fluids, one must not let them always act in isolation from each other, as is the usual practice, but successively one after another and in their proper order, on the three classes of nutritive substances. Thus: 1^0^*saliva*, on salivated or non-salivated nutrients; 2^0^*gastric juice*, on salivated nutrients with and without gastric juice; 3^0^*bile*, on nutrients treated with saliva and gastric juice with and without bile […]” (Bernard 1965b, p. 40–41). And he continued the list with the sap of the pancreas, of the intestines, of the appendix, and so on. We could also call this regime of experimentation the ‘principle of with and without,’ or the “principle of exhaustion” as I have called it elsewhere (Rheinberger, 1997).

Whereas the experimental principle just described can be seen as characterizing a certain way of doing experiments in general, we find, in the *Cahier rouge*, also remarks on the specificity of physiological experimentation, in contrast to experiments in chemistry or in physics. Mostly, experiments that follow these particular advices are simply described and not commented on. There are two entries, however, where Bernard gets explicit. In the first, under the heading of “A Physiological Principle,” he gives a reason for preferring experimentation on lower organisms over that on higher organisms: “The lower the organization, the more variety there is in less unity […] The higher the organization, the more variety there is in more unity […] It follows that experiments should be, as far as possible, conducted on lower organisms” (Bernard 1965b, p. 53). Although Bernard is best known for his experiments on mammals, he also experimented extensively with frogs and even with yeast.

The second entry is of a more technical nature and concerns the principle of experimentation in physiology itself. “Experimentation in physiology is effected by *ablation* or by *isolation*. 1^0^ by *ablation*, one seeks to see the trouble one produces in the ensemble (cutting the nerve). 2^0^ by *isolation*, one seeks to see the organ function independently of the ensemble. Examples: isolated muscles; sublingual gland.” And then he adds, without having announced it, a third point: “3^0^ by *exaggeration of the function* of the organ; section of nerves, liver, spleen […]” (Bernard 1965b, p. 172). These are three strategies of biological experimentation that actually have continued to be of relevance to this day. *Ablation* addresses the fact that by abolishing a biological entity – knocking it out — one can learn in which function it is implicated. *Isolation* is a strategy that pertains not only to biology, but it acquires a particular meaning here: Since organs, or cellular processes, are interconnected in bodies and in cells, it is often not easy to discern which function correlates with which structure. Isolation can help to solve this problem. *Exaggeration*, finally, points to a phenomenon that occupied Bernard in particular in his numerous investigations of the nervous system with its various subsystems. He found that he could not only ablate nervous actions, but in depressing one, he found that he could enhance another and make it easier to be grasped. We see thus that the *Cahier rouge* is a rich source of insight into Bernard’s experimental thinking and vivid description of the way he practiced it in his physiological laboratory setting.

## The sciences and the arts

Perhaps a most surprising aspect of these notes is Claude Bernard’s repeated allusion to the arts and the way he tries to connect these two realms of human creativity, the sciences and the arts. Early on in the *Notebook*, he states: “The human spirit proceeds in the same way in all its productions. Everywhere, in music, painting, discourses of all kinds, in the sciences as well as in the arts, there is one and the same principle for the presentation of their objects. And it is this [common] part which constitutes the *artist*.” An additional remark is to be read as a reminder to himself for working out “the art of the sciences, considered in their exposition” (Bernard 1965b, p. 36). Later in the *Cahier*, we find the following statement: “One says: This is a beautiful creation, an inspiration. An artist never knows how he arrives at things [in advance]. Even so, a scientist does not know how to find things. Once found, however, one reasons and one applies; but one needs the starting point, one needs to find where one does no longer know, for one always needs premises, and they are unknown” (Bernard 1965b, p. 135).

The “art of investigation” or “art of experimental research,” as he called it in the *Introduction* (Bernard, 1984, pp. 35, 39, 42), that is, the characterization of research as akin to an artistic process – and the other way around, looking at the arts as a research process, we might add — appears to have been constantly on Bernard’s mind. In the *Introduction*, it would take the following form. In the first chapter of its first part, he deals with the various definitions of observation and experimentation, respectively. Starting with a slogan attributed to Georges Cuvier – “the observer listens to nature, the experimenter interrogates it and forces it to reveal itself” —, he remarks that as soon as one descends into experimental practice, this clear-cut distinction starts to become blurry. And he explains: “This results, it appears to me, from having confounded the art of investigation that researches and states facts, with the art of reasoning that treats them logically in pursuit of the truth. But in investigation, one can have an activity of the mind and of the senses at the same time, be it for making observations or for doing experiments” (Bernard, 1984, pp. 34–35). “Here,” in the investigative process itself, he continues a few pages later, “we can no longer distinguish the observer from the experimenter by the nature of the research procedures applied” (Bernard, 1984, p. 42). The space of investigation, that is, defies our efforts to achieve clean distinctions, and the image of science as derived from the space of representation, where logical reasoning prevails, cannot stand in for an appropriate characterization of the research process.

## Concluding statement

This paper aimed at tracing, in a selected number of – published as well as unpublished — writings, what Claude Bernard had to say about and how he experienced the physiological laboratory, including the experimental work carried out in these spaces that were only emerging during his lifetime. The focus on the laboratory was meant to make more tangible and to develop a nuanced picture of Bernard’s epistemological position. He was staunchly opposed to metaphysics. But he also intuitively felt that in his equally staunch defense of scientific knowledge, he had to avoid a metaphysical position himself. His epistemological conviction is beautifully expressed in the following sentences with which I would like to end my parcours. They sound like an echo from Immanuel Kant’s (1999) *Critique of Pure Reason*: “We are looking for the laws of the phenomena, that is, for what is stable, invariable, permanent, eternal in them. […] We represent things in an abstractive manner in order to circumvent difficulties. Things are not rigorously such in nature, but we are obliged to conceive them in this way, and to say that one needs procedures that are nearer to the nature of things means nothing, because we do not know the nature of things, and these procedures need only to be in accord with the nature of our mind” (Bernard 1965b, p. 58).

## Footnotes

1 See the collection of the Virtual Laboratory of Physiology (https://vlp.mpiwg-berlin.mpg.de).

2 Carl Friedrich Wilhelm Ludwig (1816–1895), German physician, anatomist and physiologist.

3 Charles Adolphe Wurtz (1817–1884), French physician and chemist.

4 For the notion of exploratory experimentation, compare Burian (1997); Steinle (1997); Waters (2007).

5 As to the newer literature, compare, e.g., Wolff et al. (1967), Schiller (1967). For a more recent epistemological assessment, see Michel (1991), featuring articles by François Dagognet, Jean Gayon, Mirko Grmek, Roger Guillemin, Anne-Marie Moulin, Alain Prochiantz, Christiane Sinding, and others. See also LaFollette, Shanks (1994); Kötter (2008); Duchesneau et al. (2013). A bibliography of earlier works on Claude Bernard up to 1961 is to be found in Bernard (1961) and in Grmek (1967).

6 The term „evolutionary“ is used by Bernard not in the sense that it acquired in the aftermath of Darwin’s theory of evolution, but in the sense of the deployment of a genuinely physiological process.

7 The *Cahier rouge* was written between 1850 and 1860. It differs from earlier notebooks, in particular those used by Holmes for his book on Bernard’s early experiments, in that it generally does not record results of experiments, but rather collects ideas for future experimentation, alternating with reflexive passages on experimentation and physiology in general.

8 One is reminded here of John Stuart Mill’s “method of difference,” although in the *Introduction*, Bernard distances himself from “what the philosophers have called the method of difference” (Bernard, 1984, p. 182).
